# Thermal Stability and Non-Linear Optical and Dielectric Properties of Lead-Free K_0.5_Bi_0.5_TiO_3_ Ceramics

**DOI:** 10.3390/ma17092089

**Published:** 2024-04-29

**Authors:** Piotr Czaja, Elżbieta Szostak, Joanna Hetmańczyk, Piotr Zachariasz, Dorota Majda, Jan Suchanicz, Małgorzata Karolus, Dariusz Bochenek, Katarzyna Osińska, Jarosław Jędryka, Andriy Kityk, Michał Piasecki

**Affiliations:** 1 Institute of Technology, University of the National Education Commission, Podchorążych 2, 30-084 Krakow, Poland; 2 Faculty of Chemistry, Jagiellonian University, Gronostajowa 2, 30-387 Krakow, Poland; joanna.hetmanczyk@uj.edu.pl (J.H.); majda@chemia.uj.edu.pl (D.M.); 3 Center for Hybrid Microelectronics and LTCC, Łukasiewicz Research Network—Institute of Microelectronics and Photonics, Zabłocie 39, 30-701 Krakow, Poland; piotr.zachariasz@imif.lukasiewicz.gov.pl; 4 Department of Mechanical Engineering and Agrophysics, University of Agriculture in Krakow, Balicka 120, 31-120 Krakow, Poland; jan.suchanicz@urk.edu.pl; 5 Faculty of Science and Technology, Institute of Materials Engineering, University of Silesia in Katowice, 75 Pułku Piechoty 1a, 41-500 Chorzow, Poland; malgorzata.karolus@us.edu.pl (M.K.); dariusz.bochenek@us.edu.pl (D.B.); katarzyna.osinska@us.edu.pl (K.O.); 6 Faculty of Electrical Engineering, Czestochowa University of Technology, Armii Krajowej 17, 42-201 Czestochowa, Poland; jaroslaw.jedryka@pcz.pl (J.J.); andriy.kityk@univie.ac.at (A.K.); 7 Institute of Physics, Jan Dlugosz University, Armii Krajowej 13/15, 42-200 Czestochowa, Poland; m.piasecki@ujd.edu.pl

**Keywords:** lead-free K_0.5_Bi_0.5_TiO_3_ ceramics, ferroelectric materials, dielectric permittivity, Raman, FT-FIR, FT-MIR spectroscopies, DFT calculations, SHG technique

## Abstract

Lead-free K_0.5_Bi_0.5_TiO_3_ (KBT) ceramics with high density (~5.36 g/cm^3^, 90% of X-ray density) and compositional purity (up to 90%) were synthesized using a solid-state reaction method. Strongly condensed KBT ceramics revealed homogenous local microstructures. TG/DSC (Thermogravimetry-differential scanning calorimetry) techniques characterized the thermal and structural stability of KBT. High mass stability (>0.4%) has proven no KBT thermal decomposition or other phase precipitation up to 1000 °C except for the co-existing K_2_Ti_6_O_13_ impurity. A strong influence of crystallites size and sintering conditions on improved dielectric and non-linear optical properties was reported. A significant increase (more than twice) in dielectric permittivity (*ε*_R_), substantial for potential applications, was found in the KBT-24h specimen with extensive milling time. Moreover, it was observed that the second harmonic generation (λ_SHG_ = 532 nm) was activated at remarkably low fundamental beam intensity. Finally, spectroscopic experiments (Fourier transform Raman and far-infrared spectroscopy (FT-IR)) were supported by DFT (Density functional theory) calculations with a 2 × 2 × 2 supercell (*P*4_2_*mc* symmetry and C4v point group). Moreover, the energy band gap was calculated (*E*_g_ = 2.46 eV), and a strong hybridization of the O-2*p* and Ti-3*d* orbitals at *E*_g_ explained the nature of band-gap transition (Γ → Γ).

## 1. Introduction

Ferroelectrics and relaxor ferroelectrics are materials that exhibit high electrostriction. This functional materials group is becoming valuable for wide technological applications such as transducers, multilayer capacitors, micromechanical systems, pyroelectric detectors, and electro-optical switches [1,2,3,4,5,6,7,8,9,10].

Unfortunately, conventionally implemented ferroelectrics, PbZrO_3_-PbTiO_3_ (PZT), (Pb,La)(Zr,Ti)O_3_ (PLZT), and Pb(Mg_1/3_Nb_2/3_)O_3_ (PMN), comprise toxic lead and according to the obligatory EU directives [11,12,13], it is necessary to limit the harmful elements released into the environment. Therefore, comprehensive development of modern lead-free ferroelectrics and relaxors with comparable or even more beneficial properties than lead-based materials is required [1,2,4,5,7,8,9,10].

Among a plurality of synthesized lead-free materials such as barium titanate (BaTiO_3_) [14,15,16,17], lithium niobate (LiNbO_3_) [18,19,20], potassium sodium niobate (K_0.5_Na_0.5_NbO_3_) [21,22,23,24], strontium titanate (SrTiO_3_) [25,26,27], and sodium bismuth titanate (Na_0.5_Bi_0.5_TiO_3_) [28,29,30,31,32,33,34,35,36], extraordinary attention has been focused on potassium bismuth titanate (K_0.5_Bi_0.5_TiO_3_) [36,37,38,39,40,41,42,43,44,45,46,47,48,49,50,51,52]. The latter material is characterized by a relatively high Curie temperature *T*_C_ ≈ 380 °C [36,51] and a large depolarization temperature *T*_d_ ≈ 285 °C [52], which destines K_0.5_Bi_0.5_TiO_3_ (KBT) materials to be competitive with currently used ferroelectrics such as bismuth titanate (Bi_4_Ti_3_O_12_, *T*_C_ ≈ 675 °C) [53] or lead titanate (PbTiO_3_, *T*_C_ ≈ 490 °C) [54].

As previously shown [40], bulk density is the most crucial factor for polycrystalline KBT ceramics. However, KBT density may be underestimated and restrict the ferroelectric properties [40,41,42,43,44,55] due to the formation of impurity phases in ceramics sintering. Therefore, KBT manufacturing with the lowest possible impurity phase content is imperative [45,46,47,48,49,56].

The present work aimed to refine the synthesis of pure high-density KBT by adjusting the milling time. In addition, the infrared and Raman spectroscopy results were compared with the DFT calculations (CASTEP code) to explain the effect regarding KBT dielectricity depending spontaneously on morphology. Finally, the second-harmonic signal was generated in pure KBT to reveal distinctive non-linear optical properties.

## 2. Materials and Methods

### 2.1. K_0.5_Bi_0.5_TiO_3_ Preparation

According to the chemical reaction:

0.25 K_2_CO_3_ + 0.25 Bi_2_O_3_ + TiO_2_ → (K_0.5_Bi_0.5_)TiO_3_ + 0.25CO_2_ ↑ (1)

lead-free polycrystalline potassium bismuth titanate (KBT) was prepared by a solid-state synthesis route. For this purpose, high-purity titanium (IV) oxide TiO_2_ (99.0%, Sigma Aldrich, St. Louis, MO, USA), bismuth (III) oxide Bi_2_O_3_ (99.9%, Acros Organics, Geel, Belgium), and potassium carbonate K_2_CO_3_ (cz.d.a., Chempur, Piekary Śląskie, Poland) were weighed in stoichiometric proportions utilizing an AS 310.R2 (Radwag, Radom, Poland) analytical balance. The K_2_CO_3_ precursor was annealed at 210 °C for 3 h before KBT synthesis for moisture removal according to preliminary TG analysis (Supplementary Materials).

**Figure 1:**
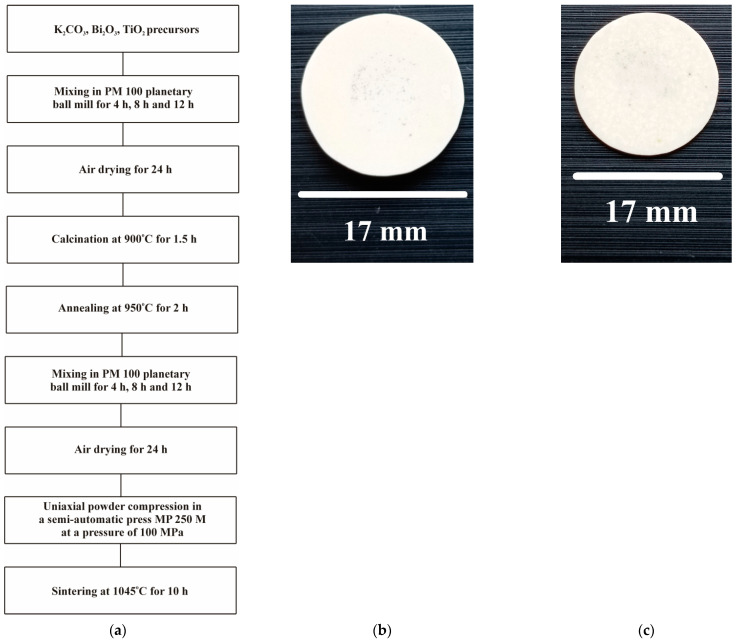
Block diagram of KBT manufacturing (**a**) and photos of KBT-8h (**b**) and KBT-24h (**c**) pellets.

A complete technological process is shown in Figure 1a, while some KBT pellets are presented in Figure 1b (KBT-8h) and Figure 1c (KBT-24h). Firstly, the precursors were milled in the PM 100 (Retsch, Haan, Germany) planetary mill with 5 mm yttrium-doped zirconia (YTZ) balls as the milling medium. The powder mixture was then uniaxially compressed on a semi-automatic MP 250 M press under a pressure of 100 MPa. Next, the specimens were calcined in an FCF 4/160 M (Czylok, Jastrzębie Zdrój, Poland) chamber furnace with a temperature control of ±1 °C (MRT—4 type regulator). After calcination, the specimens were crushed and ground in a planetary ball mill. Then, powders were dried, pressed into pellets, and sintered at 1045 °C for 10 h. Therefore, starting now, KBT-8h, KBT-16h, and KBT-24h refer to specimens with a total milling time of 8 h (2 × 4 h), 16 h (2 × 8 h), and 24 h (2 × 12 h), respectively.

The sintered KBT ceramics were slightly translucent and ivory in color. The apparent density of around 5.36 g/cm^3^ was estimated by hydrostatic weighing (Archimedes principle). Initial strength tests involving the ultrasonic method described elsewhere [57] revealed suitable mechanical properties (*E*~100 MPa, *G*~39 MPa) of KBT. Both elasticity parameters (Young and Kirchoff modules) corresponded to previously reported findings [50], and the slight discrepancies, despite identical processes, were due to diverse substances, i.e., Bi_2_O_3_ and K_2_CO_3_.

### 2.2. SEM/EDXS (Scanning Electron Microscopy/Energy-Dispersive X-ray Spectrometry) Investigations

Microstructure analysis was completed on the JSM-7100F microscope (Jeol Ltd., Tokyo, Japan) with a thermal field emission (T-FE) electron gun. A modern electro-optic column system provided high-quality and -resolution SEM images, while a JEOL-EDXS (Jeol Ltd., Tokyo, Japan) (X-ray energy dispersive) spectrometer estimated the elemental compositions. The EDXS data were collected and averaged across 5 points for each specimen.

### 2.3. X-ray Diffraction

X-ray diffraction measurements were taken using an Empyrean PANalytical diffractometer operating in the Bragg–Brentano geometry with the Cu radiation (λ_Kα_ = 1.5418 Å) and PIXcell. The structural characterization was performed using the High Score Plus PANalytical software 3.0 based on the Williamson–Hall theory [58] and the Rietveld method [59,60,61,62]. Finally, phase analysis was performed using the crystallographic database ICDD PDF4+ 2016. The X-ray data were collected in the angular range of 10° < 2*θ* < 150°.

### 2.4. Thermogravimetry and Differential Scanning Calorimetry

Thermogravimetric (TG) curves were recorded using a Mettler Toledo TGA/SDTA 851^e^ (Mettler Toledo, Greifensee, Switzerland) instrument calibrated with indium, zinc, and aluminum standards. The experiment was carried out in a 25–1000 °C temperature range with a heating rate of 10 °C/min. During the measurements, specimens were held in an alumina crucible with an airflow of 60 cm^3^/min.

In turn, differential scanning calorimetry (DSC) was carried out utilizing a Mettler Toledo DSC 822^e^ apparatus (Mettler Toledo, Greifensee, Switzerland) equipped with a liquid nitrogen cooling system. The heat flux and temperature calibration were performed using indium and zinc standards for specimens weighing around 3 mg, placed in sealed aluminum pans. The empty pan was used as DSC reference data. In the DSC experiment, the specimens were heated to 600 °C at 10 °C/min, the isothermal phase was held for 1 min, and then the specimens were cooled to ambient conditions at 10 °C/min. All measurements were completed at an argon flow of 60 cm^3^/min.

### 2.5. Vibrational and Raman Spectroscopy

The far-infrared (FT-FIR; 400–50 cm^−1^) and mid-infrared (FT-MIR; 4000–400 cm^−1^) investigations were performed using a Bruker VERTEX 70v (Bruker Optic GmbH, Ettlingen, Germany) FT-IR instrument with a spectral resolution of 2 cm^−1^. FT-FIR spectra were recorded for specimens suspended in Apiezon grease (Apiezon N, St. Louis, MO, USA) on a polyethylene window, while FT-MIR spectra were collected for compressed KBr pellets and examining materials. A DE-202A helium cryostat (Advanced Research System, Macungie, PA, USA) and water-cooled ARS-2HW helium compressor operating in a closed cycle manner were employed to gain the low-temperature infrared spectra (15 ÷ 295 K) with thermal stabilization of ±0.2 K.

On the other hand, the vibrational spectra (FT-RS) were measured by a MultiRAM FT–Raman instrument (Bruker Optic GmbH, Ettlingen, Germany) equipped with the 1064 nm laser line and germanium detector. The Raman spectra were collectively gathered (32 scans) at room temperature in a wavenumber range of 4000–50 cm^−1^ with a spectral resolution of 4 cm^−1^.

### 2.6. Dielectric Measurements

A modern LCR-8110G meter (GW Instek, New Taipei, Taiwan) was used to study the dielectric properties of KBT ceramics. The electrochemical impedance spectroscopy (EIS) was measured in a 25–600 °C temperature range, and the frequency varied from 1 kHz to 2 MHz. First, square polycrystalline specimens of 2 mm were cut using a WS-10 wire saw. Then, the KBT slabs were purified in a 20 mL vessel with acetone for 15 min. After drying, the silver electrodes were sputtered on the KBT specimens and fired at 850 °C for 30 min.

### 2.7. DFT Calculations

The band structure and total and partial density of states for KBT ceramics were calculated. Next, theoretical infrared and Raman spectra were simulated using the density functional perturbation theory implemented in the CASTEP (CAmbridge Serial Total Energy Package) code [63,64]. The KBT unit cell geometry optimization preceded the DFT (Density Functional Theory) calculation, where the crystal structure described by Smazhevskaya et al. [65] was adopted as a starting point for the computational data.

The K_0.5_Bi_0.5_TiO_3_ compound crystallizes in tetragonal symmetry (*P*4*mm* space group, No. 99) with cell parameters *a* = 3.9388 Å and *c* = 3.9613 Å [66]. The Bi and K atoms were statistically distributed at the A position in the ABO_3_ structure with identical occupancy factors. Therefore, a supercell 2 × 2 × 2 of *P*4_2_*mc* symmetry (No. 105, point group *C*_4*v*_) with the cell parameters *a* = 7.88 Å and *c* = 7.92 Å was constructed to simulate the atom disorder. During geometry optimization, the ion positions were relaxed while the unit cell parameters remained constant. The PBE (Perdew–Burke–Ernzerhof) functional form was based on a numerical GGA (generalized gradient approximation) model for the exchange and correlation energies of atoms [67,68]. The GGA pseudopotential describes equilibrium geometry quite well but may slightly underestimate the KBT energy gap. The norm-conserving pseudopotential [63] was utilized with a plane-wave cut-off energy of 1100 eV. The Brillouin zone k-point grid of 2 × 2 × 2 was sampled using the Monkhorst Pack scheme [69,70]. The convergence threshold for self-consistent iterations was adopted at 10*^−^*^10^ eV/atom. The limits for optimization were set at the following levels: maximum energy change 5·10^−7^ eV/atom, maximum displacement tolerance 5·10^−4^ Å, maximum force 0.01 eV/Å, and maximum stress 0.02 GPa.

The Raman activity tensors were evaluated using a hybrid DFPT/finite displacement approach [71]. Phonon wavenumbers were extracted by diagonalizing the dynamical matrices computed from the DFPT method [64]. A dispersion of phonons was calculated for *k* = 0 and high-symmetry directions of the Brillouin zone.

The computed Raman activities were numerically transformed into theoretical Raman intensities by the following expression:

*I_i_* = 10^−12^·(*ν*_o_ − *ν_i_*) *RA_i_*, (2)

where *I_i_*—the Raman peak intensity, *RA_i_*—the Raman scattering activity, *ν_i_*—the wavenumber of the normal mode, and *ν*_o_ = 939.8 cm^−1^—the light wavenumber of the excitation laser [72]. Then, theoretical intensities were convoluted with the Lorentzian function, and the width of vibrational bands was matched to experimental spectra.

### 2.8. Second-Harmonic Generation

**Figure 2:**
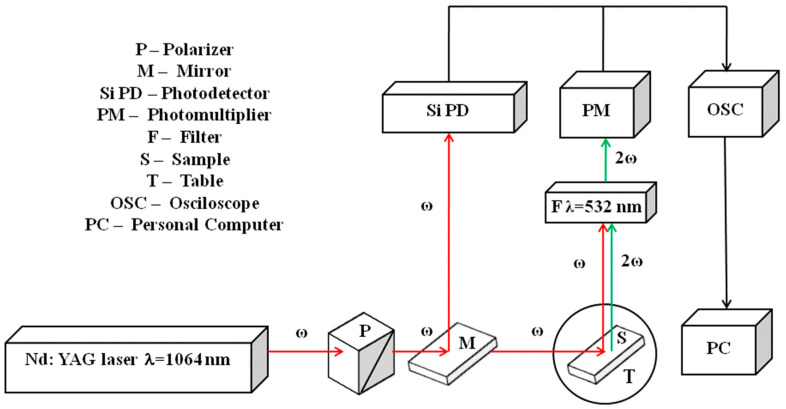
Experimental setup to generate the second-harmonic light.

The measuring system for the higher-harmonics investigation (2nd and 3rd) is shown in Figure 2. An 8-nanosecond pulsed laser Nd:YAG with a 1064 nm wavelength and frequency repetition of 10 Hz was used as the excitation source. The Glan–Taylor polarizer of high laser damage resistance (4 GW/cm^2^ @ 1064 nm) tuned the laser beam power up to 100 J/m^2^.

The optical setup was covered with a non-transparent segment annihilating external light sources. A chopper was also mounted behind the sample holder to eliminate internal beam reflections from chamber walls.

A non-linear optical (NLO) crystal β-BBO (barium borate) was used as a reference. A Si photodetector evaluated the fundamental radiation, while the SHG signal detected in reflected geometry was further processed by a Hamamatsu photomultiplier equipped with an interferometer filter working at 532 nm. A Tektronix MSO3054 oscilloscope (Tespol sp. z.o.o., Wrocław, Polska) with a 2.5 GS/s sample rate was used for data visualization. In addition, an SV2100 K-MAC (Korea Materials and Analysis Corp., Daejeon, Republic of Korea) compact spectrometer was operated for SHG spectral analysis.

## 3. Results and Discussion

### 3.1. Thermal Stability of KBT

The preliminary study of precursors preceded the technological approach to KBT manufacturing. In particular, the thermogravimetric analysis of K_2_CO_3_ was considered due to a relatively low decomposition temperature of potassium carbonate. In addition, the phase purity of precursors was also examined using the X-ray diffraction technique (see Figures S1 and S2 in Supplementary Materials).

**Figure 3:**
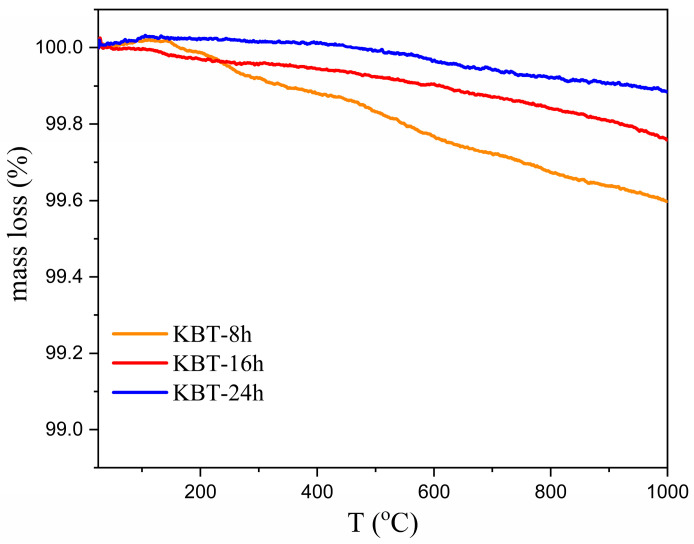
The weight loss analysis of KBT at high temperatures.

The thermal and structural stabilities of KBT were investigated to yield an overview of industrial applicability. Thermogravimetric curves (TGs) confirm mass stability over the broad temperature range (Figure 3). The highest weight loss for KBT-8h does not exceed 0.4%, and the longer the milling time, the more insignificant mass decline (0.2% for KBT-24h). Therefore, it can be concluded that extended milling increases the contact surface of particles and the uniformity of their spatial distribution. This leads to a more homogeneous and thermally stable local microstructure of KBT ceramics.

**Figure 4:**
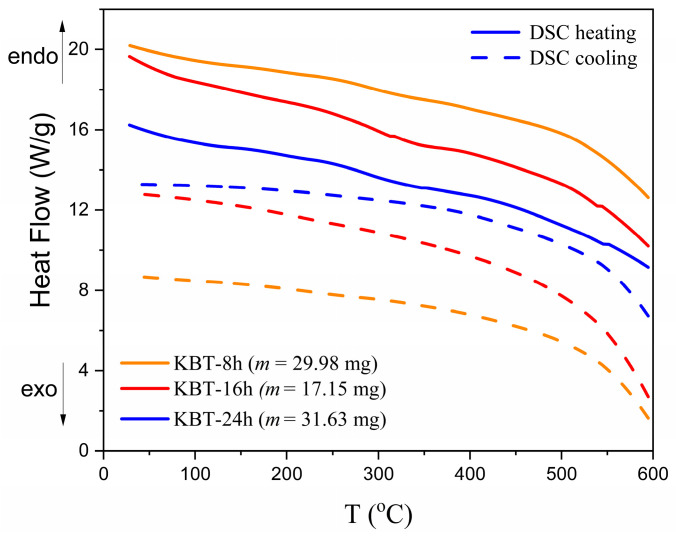
DSC curves in heating (solid lines) and cooling (dash lines) regimes registered for KBT ceramics.

Similarly, DSC analysis reveals no distinct anomalies in heat flow (Figure 4), indicating KBT decomposition or precipitation of different impurity phases. All DSC curves exhibit similar characteristics of a slow decline in heat flow rate at high temperatures, showing that grain size composition may be essential for KBT ceramics. Therefore, attention was paid to the influence of milling time on KBT physicochemical properties.

### 3.2. Morphology of KBT Ceramics

Volumetric mass density revealed a negligible influence of milling time on the ρ parameter. For the KBT-8h, KBT-16h, and KBT-24h specimens, the bulk densities were 5.359 g/cm^3^, 5.367 g/cm^3^, and 5.368 g/cm^3^, which is approximately 90 ± 1% of the X-ray density (5.96 g/cm^3^) previously reported [50]. The preceding results show the desired tendency: the longer the milling time, the more condensed the KBT ceramics.

**Figure 5:**
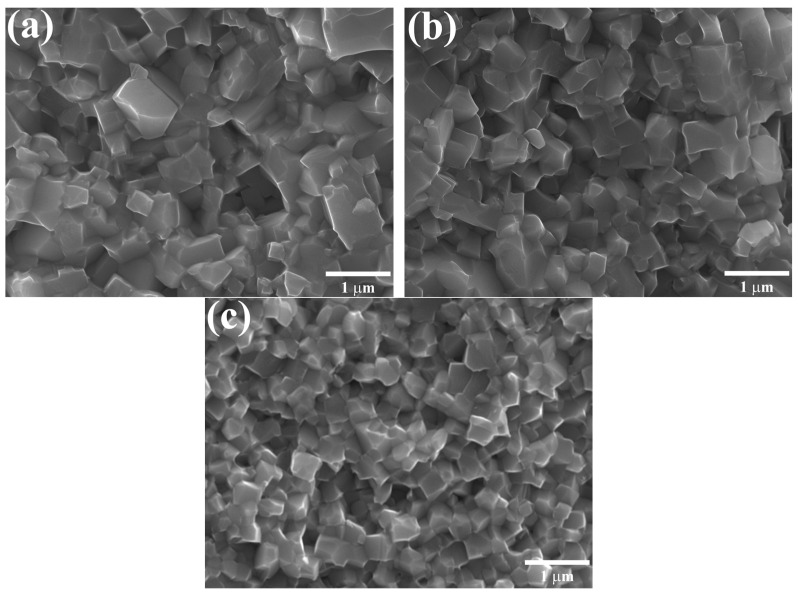
SEM micrographs of (**a**) KBT-8h, (**b**) KBT-16h, and (**c**) KBT-24h.

Figure 5 shows the local microstructures of the KBT compounds sintered at 1045 °C to minimize impurity phases. All KBT specimens were characterized by well-defined, irregular grains with sharp and precise edges ranging from 0.25 to 1 µm. A detailed SEM analysis confirms that extended milling composes the more homogeneous local microstructure with numerous diminutive crystallites, resulting in a higher density of KBT ceramics.

**Figure 6:**
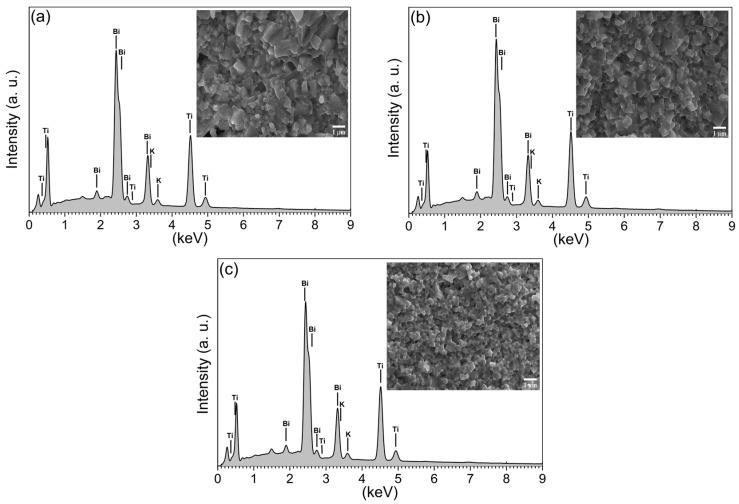
EDXS spectra corresponding to (**a**) KBT-8h, (**b**) KBT-16h, and (**c**) KBT-24h.

The EDXS analysis was performed at five randomly selected sample positions and averaged to confirm the KBT stoichiometry. Figure 6 presents the selected energy-dispersive spectra for KBT specimens, where the intensity of the spectral lines and individual element contents seem similar.

However, the outlined compositional data reveal some discrepancies in the KBT stoichiometry. For example, Table 1 shows an excess of Bi over Ti and K of around 17% and 20%, respectively. Thus, in the EDXS inspection area, in addition to KBT crystallites, at least one Bi-rich phase should be present. Therefore, to discover the nature of this phenomenon, structural studies were carried out to exclude or confirm the formation of impurity phases.

### 3.3. The X-ray Measurements

**Figure 7:**
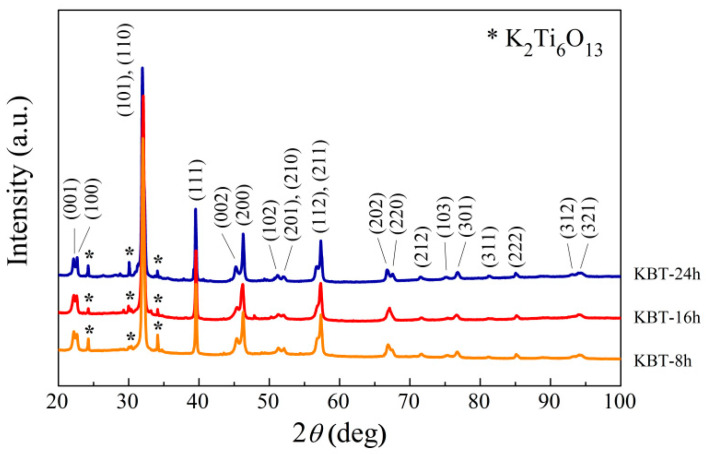
X-ray diffraction patterns of KBT ceramics at room temperature.

X-ray diffraction patterns for KBT are presented in Figure 7. A perovskite structure (*P*4*mm* symmetry) reported in the ICDD PDF4+ database (reference no. 01-072-8121) was recognized for all specimens [43]. The relatively narrow and well-defined Bragg reflexes of the K_0.5_Bi_0.5_TiO_3_ phase indicate an appropriate synthesis of KBT ceramics.

The KBT diffraction patterns shown in previous reports [43,44] indicated some impurity phases, such as K_2_Ti_6_O_13_ (ICDD PDF4+: 04-011-1358) or K_2_Ti_8_O_17_ (ICDD PDF4+:04-009-4964). Thus, as expected, 80–90 wt% of K_0.5_Bi_0.5_TiO_3_ (ICDD PDF4+: 01-072-8121) and a small amount (10–20 wt%) of the K_2_Ti_6_O_13_ phase (ICDD PDF4+; 00-040-0403) were detected [43,44].

The refined cell parameters and crystallite sizes of the dominant phase (K_0.5_Bi_0.5_TiO_3_) are summarized in Table 2. The extended milling time slightly elongates the *P*4*mm* unit cell in the *c*-axis direction compared to theoretical values (*a* = 3.9388 Å and *c* = 3.9613 Å); therefore, there is a tendency for the *c*/*a* ratio and unit cell volume to increase.

The Rietveld analysis, which is an integral part of the High Score Plus PANalytical software 3.0, showed refinement parameters of *R*_exp_ = 10% and *R*_B_ = 16%, respectively.

Simultaneously, a significant reduction in the mean KBT crystallite size from nearly 400 Å to about 300 Å was observed with milling time (Table 2). Moreover, the lattice strains for KBT-8h, KBT-16h, and KBT-24h were evaluated in the 0.1–0.3% range.

### 3.4. Optimization of KBT Structure for Vibrational Spectra Calculations

**Figure 8:**
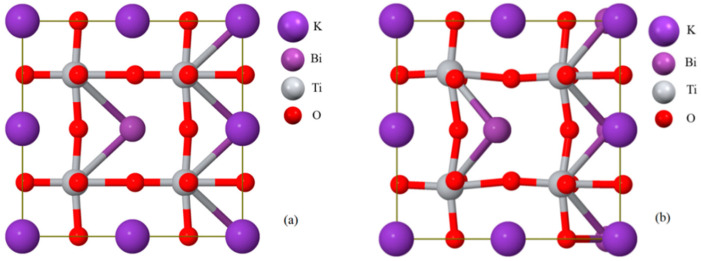
The 2 × 2 × 2 supercell models of KBT ceramics: (**a**) initial geometry and (**b**) equilibrium state after DFT structure optimization.

The K_0.5_Bi_0.5_TiO_3_ compound crystallizes in a tetragonal system with *P*4*mm* symmetry (No. 99). The space group of the 2 × 2 × 2 supercell adopted for DFT calculations was *P*4_2_*mc* (No. 105) with a *C*_4v_ (4*mm*) point group, where the 40 constituent atoms (Figure 8) occupy the crystallographic positions (Wyckoff notation) listed in Table 3. According to group theory [73,74], the 120 irreducible representations of vibrational modes for the *P*4_2_*mc* system can be given by the following formula:

*Γ* = 18*A*_1_ + 10*A*_2_ + 18*B*_1_ + 10*B*_2_ + 32*E*. (3)

For a plurality of vibrations at the *Γ* point (*E* modes are two-fold degenerate), a factor group analysis predicts the 117 active optical and 3 acoustic (phonon) modes. Among the 117 optical representations, the *A*_2_ modes are completely inactive at infrared and Raman spectroscopies. Therefore, 17*A*_1_ + 31*E* active IR modes and 17*A*_1_ + 18*B*_1_ + 10*B*_2_ + 31*E* active Raman modes can be distinguished. The wavenumbers for 120 vibrational modes are listed in Table S1 (see Supplementary Materials).

Comparing both crystallographic structures (Figure 8), one can easily conclude that the surroundings change significantly for Bi and Ti atoms inside the *P*4_2_*mc* unit cell. Hence, no unit cell distortions of KBT were observed on the X-ray diffraction patterns. However, some anomalies in optical vibrational spectroscopy should be expected due to the displacement of internal atoms.

Table 4 presents selected bond lengths and bond angles of KBT ceramics corresponding to the pristine local environment and the relaxed structure (DFT calculation) at the equilibrium state.

The FT-FIR and FT-MIR spectra temperature evolution is shown in Section 3.6. However, the infrared data recorded at room temperature are referenced here to compare the experimental results with the DFT calculations. Also, the DFT computational results verified the Raman vibration spectrum collected at ambient conditions.

**Figure 9:**
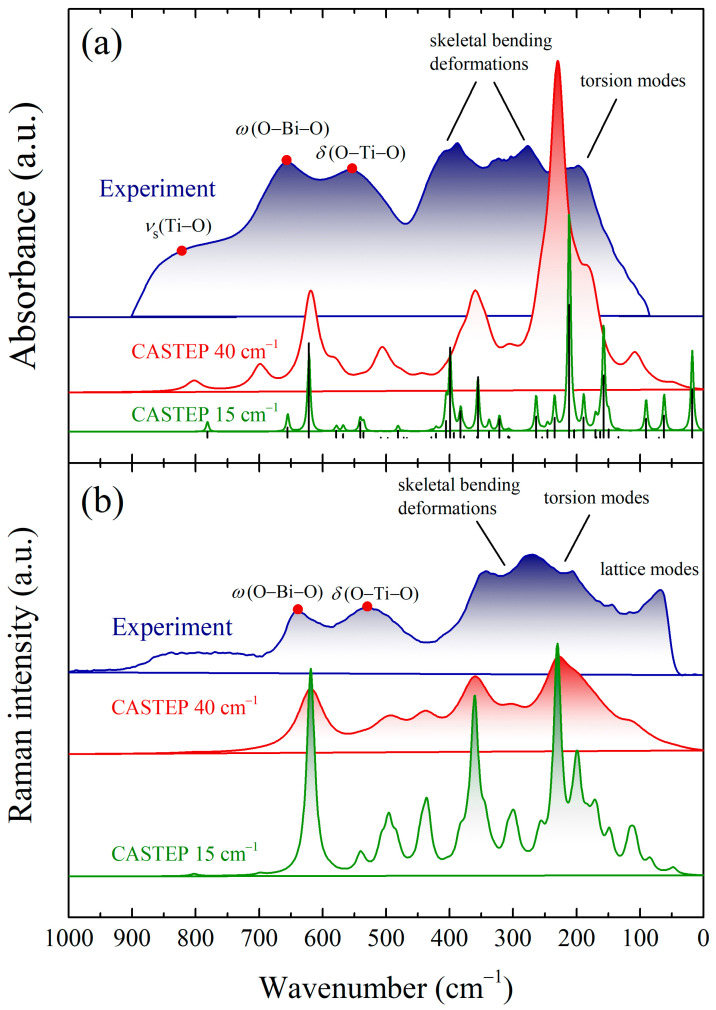
Experimental (**a**) FT-IR and (**b**) Raman spectra of KBT-24h compared with DFT calculations.

A comparison of the calculated FT-IR and Raman spectra using periodic boundary conditions (PBCs) and experimental data is presented in Figure 9. The physical meaning of vibrational modes was established due to previous reports [75,76] and the *Jmol* software (version 16.1.47) was used to visualize the CASTEP calculations [77]. One can see a satisfying agreement between vibrational modes calculated at the *Γ*-point and observable peaks in the experiment. Nevertheless, several simulated and experimentally detected vibration modes diverge on the wavenumber scale due to a slight difference in local atom environments.

As shown in Figure 9a, three extensive IR bands are located between 450 and 900 cm^−1^. A broad low-intensity peak with a maximum of 819 cm^−1^ refers to the symmetric stretching mode *ν*_s_(Ti–O) of the TiO_6_ octahedrons surrounding the bismuth ion. The influential IR peak in the supercell model located at 618 cm^−1^ was assigned to the symmetrical stretching *ν*_s_(O–Ti–O) vibrations. However, this absorption band was downshifted by 40 cm^−1^ compared to the experimental data. Moreover, the vibrational bands in a wavenumber region of 600–400 cm^−1^ can be assigned to the symmetric and asymmetric stretching modes ν_s_(Ti–O–Ti), ν_as_(Ti–O–Ti) and the bending deformation mode δ(O–Ti–O).

On the other hand, the wagging modes correspond to vibrational bands at 656 and 638 cm^−1^ at IR and RS spectra, respectively. The infrared peak at 564 cm^−1^ is related to the asymmetric stretching vibrations *ν*_as_(O–Ti–O), whereas the Raman peak at 528 cm^−1^ (Figure 9b) is attributed to the bending vibrations *δ*(O–Ti–O) of the octahedral TiO_6_ groups.

In turn, both IR and RS spectra below 400 cm^−1^ show vibrational bands related to skeletal bending deformations with overlapping bending deformation modes *δ*(O–Ti–O). Finally, torsion modes were observed in the spectral region of 220–140 cm^−1^, and an explicit maximum at 142 cm^−1^ was related to lattice vibrations *ν*_L_.

### 3.5. Band Structure of KBT

**Figure 10:**
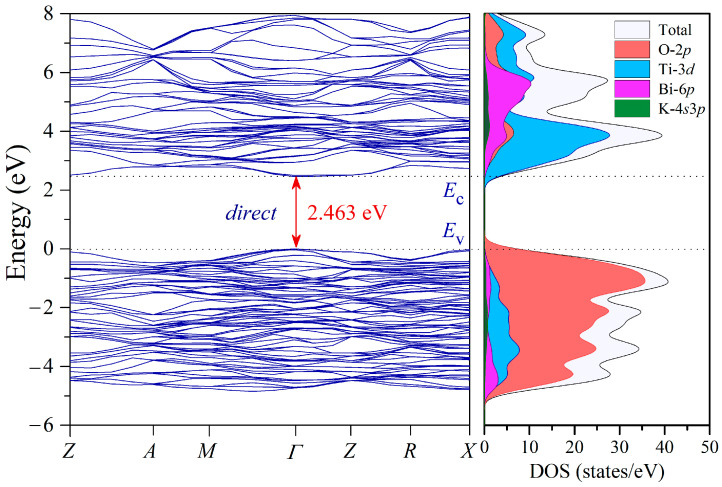
Electronic band structures (**left panel**) and partial density of states (**right panel**) of Ti, O, Bi, and K orbitals for KBT ceramics.

The KBT electronic properties were investigated using the CASTEP code. The high-symmetry *k*-path in the Brillouin zone used for the DFT computations of the KBT electronic band structures was as follows: *Z*(0, 0, 1/2), *A*(1/2, 1/2, 1/2), *M*(1/2, 1/2, 0), *Γ*(0, 0, 0), *Z*(0, 0, 1/2), *R*(0, 1/2, 1/2), *X*(0, 1/2, 0). Figure 10 shows the calculated electronic band structures and partial density of states (PDOSs).

In the highest valence band (Figure 10), a contribution of the O-2*p* orbitals is predominant, while the densities of Ti-3*d* and Bi-6*p* states are modest and even negligible for K-4*s*3*p*. Moreover, the lowest conduction band spanning the energy range from 2.46 eV to 8.00 eV consists mainly of Ti-3*d* states with a humbler contribution of the O-2*p* and Bi-6*p* orbitals. Therefore, a strong hybridization of the O-2*p* and Ti-3*d* orbitals is observed near the gap energy (*E*_g_). However, a much weaker hybridization is expected between the O-2*p* and Bi-6*p* orbitals, as their PDOS contribution to the valence and conductive bands near *E*_g_ is inferior.

The above results indicate that KBT ceramics reveal a direct electronic band gap transition (*Γ* → *Γ*) at a level of 2.463 eV [78], which reasonably agrees with the experimental data [79].

### 3.6. FT-MIR and FT-FIR Investigations

**Figure 11:**
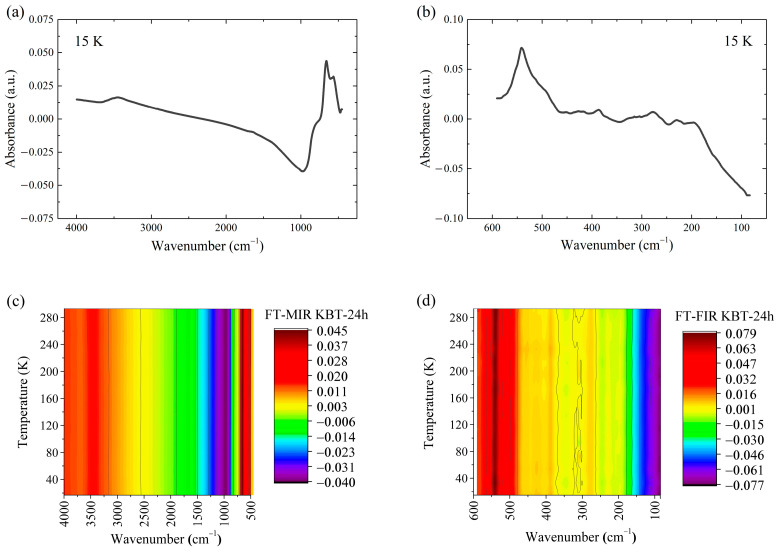
FT-MIR (**a**,**c**) and FT-FIR (**b**,**d**) spectra of KBT-24h and their temperature evolution.

The infrared spectra were collected in a comprehensive energy range to evaluate the low-temperature effect on KBT’s vibrational structure. Figure 11 shows the FT-MIR and FT-FIR spectra of KBT-24h at 15 K and their thermal evolution. Other KBT specimens revealed similar vibrational characteristics.

As shown in Figure 11, the mid-infrared spectra are composed of three (818, 656, and 564 cm^−1^), and the far-infrared spectra of five (540, 389, 275, 228, and 195 cm^−1^), original vibration bands of KBT ceramics. A detailed description is provided in Table S1 (see Supplementary Materials).

Figure 11 predicts the structural and functional stabilities of the KBT material also at low temperatures. The broad low-intensity peak at 3450 cm^−1^ was attributed to the asymmetric *ν*_as_(H–O) and symmetric *ν*_s_(H–O) stretching modes of the water molecules adsorbed on the ceramic surface [80,81].

### 3.7. Dielectric Permittivity Measurements

**Figure 12:**
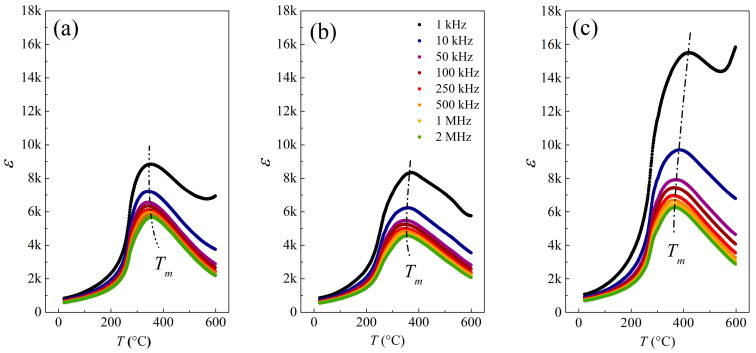
Relative permittivity for (**a**) KBT-8h, (**b**) KBT-16h, and (**c**) KBT-24h.

The relative permittivity (*ε*_R_) and loss tangent (tan*δ*) parameters were investigated to evaluate KBT’s dielectricity. All of the specimens reveal increased *ε*_R_ and strongly diffused ferro- to paraelectric transition with maximum permittivity (*ε*_m_) at the *T*_m_ points (Figure 12).

The grain size usually affects the dielectric properties of ceramic ferroelectrics by, e.g., interfacial stresses, porosity level, or grain boundaries [82,83]. Therefore, KBT-24h encloses the highest crystallite homogeneity due to the most extended grinding and, consequently, the largest permittivity concerning KBT-8h or KBT-16h. Indeed, one can see that *ε*_m_(*T*_m_) is shifted towards higher temperatures for extended milling (Figure 12). At a characteristic frequency of 50 kHz, *T*_m_ is equal to 343, 352, and 374 °C for KBT-8h, KBT-16h, and KBT-24h, respectively.

Moreover, an EIS examination indicated the *ε*_R_ dispersion simultaneously without the *ε*_m_ shifting towards higher temperatures with frequency, which is characteristic of ferroelectrics. In general, ferroelectrics reveal high permittivity at low frequencies; however, the Maxwell–Wagner two-layer model [84] describes a systematic decrease in *ε*_R_ with frequency, where high conductivity is attributed to crystallites and high resistivity to grain boundaries. Also, in low-frequency regions and with an applied external electric field, the migration of carriers from crystallite interiors to the grain boundary regions arises efficiently, contributing to the high dielectric constant. In contrast, at higher frequencies, the mobility of electric charges is limited by rapid polarization changes, resulting in charge deficiency at the grain boundary regions and lower permittivity [84,85,86].

Interestingly, a slight variation in relative permittivity within the device operating in the *f*-range is desired in practical applications. For example, ferroelectric capacitors commonly show non-linear dependence on temperature and voltage but also reveal massive loss tangents, which are all challenging in technical implementations.

**Figure 13:**
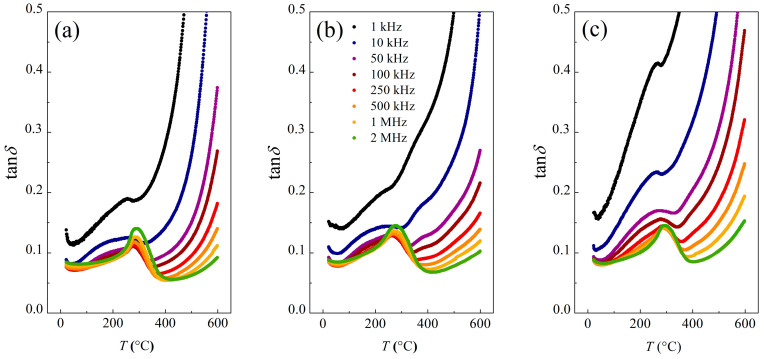
Dielectric loss (tan *δ*) of (**a**) KBT-8h, (**b**) KBT-16h, and (**c**) KBT-24h.

Figure 13 presents the dielectric loss factor (tan*δ*) for KBT as a function of temperature and frequency. One can see a similarity for all KBT specimens, i.e., a more or less pronounced tan*δ* maximum at around 280 °C. It was found that, for KBT-8h and KBT-16h, tan*δ* shows a rapid increase above 250 °C at 1 kHz and 10 kHz, and for KBT-24h, this effect is even noticeable around 40 °C.

A congruent behavior was found at higher frequencies, of 50 kHz–2 MHz (Figure 13). Behind the peak around 280 °C, the loss tangent factor reaches the local minimum at 400 °C, which is related to *ε*_m_, and there is a further rapid rise of tan*δ* above 400 °C from a substantial increase in electric conductivity.

The loss tangent factor is increased at low frequencies (Figure 13) since the strongly accumulated electric carriers at highly resistive grain boundaries require raised excitation energy. On the other hand, at high-frequency regions, less excitation energy is needed at boundary regions, resulting in small dielectric losses [57], as described by the Koop two-layer model [84].

A modest tan*δ* value and its minor variability over a wide temperature range are expected in technological aspects. KBT ceramics satisfy the above restrictions in the 50 kHz–2 MHz frequency range and temperatures from 20 to 200 °C.

Further dielectric data analysis reveals that only at room temperature and for 1 kHz is the relative permittivity of KBT-16h higher than KBT-8h (Table 5). On the other hand, a significant increase in dielectric permittivity was observed for KBT-24h. For example, *ε*_RT_ increases at 1 kHz by 27 and 24% compared to KBT-8h and KBT-16h, respectively. In turn, a more significant *ε*_m_ increase for KBT-24h of 76 and 86% concerning KBT-8h and KBT-16h, respectively, was detected.

Similar behavior was also observed for other frequencies, indicating the influence of KBT morphology on its dielectric properties. Thus, a lower concentration of closed (internal) pores due to appropriate milling of grains enforces preferable dielectric properties. The permanently weaker *ε* for KBT-8h and KBT-16h is probably due to heterogeneity in crystallite sizes resulting from insufficient milling.

Table 5 compiles the results for KBT ceramics [45,49] and other commonly used ferroelectrics [87,88] in terms of the maximal relative permittivity (*ε*_m_) and relative permittivity at ambient conditions (*ε*_RT_). One can conclude that excellent dielectric properties characterize the KBT-24h specimen regarding the previously reported lead-bearing PbTiO_3_ material, lead-free Na_0.5_Bi_0.5_TiO_3_ ceramic, and even the K_0.5_Bi_0.5_TiO_3_ analogue [45,49]. Interestingly, a significant improvement in the KBT dielectricity was gained by modifying one technological parameter (milling time).

Several studies were issued regarding the effects of technological conditions on selected physicochemical properties of manufactured materials. For example, an exciting article concerning the mechanosynthesis of lead-free K_0.5_Na_0.5_NbO_3_ ceramics was recently published [88], where only milling time was adjusted in the technological process. A high-energy mill was used for K_0.5_Na_0.5_NbO_3_ synthesis [88], while in the present work, a significant improvement in dielectric properties was gained by using the planetary ball mill. This is important since the ferroelectrics industry should conform to synthesis processes for the large-scale production of lead-free materials.

### 3.8. Optical Properties

**Figure 14:**
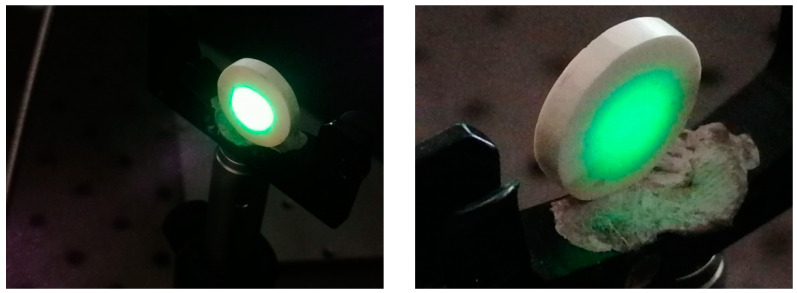
Bright-greenish spots (visible electromagnetic spectrum segment) as an optical response to radiation excitation with 1064 nm (N-IR) for KBT-8h.

A fundamental laser beam of near-infrared wavelength (λ_N-IR_ = 1064 nm) was focused on KBT pellets (Figure 14) to determine whether the KBT ceramics are excited by electromagnetic radiation and generate the second-harmonic light. Indeed, the SHG wave was produced by all KBT specimens in the visible light range (λ_SHG_ = 532 nm) as intense greenish glows.

It is also interesting that SHG light was emitted from both sides of the KBT specimens (Figure 14). Thus, a substantial volume of KBT ceramics (irradiated area) were entangled in SHG wave generation.

**Figure 15:**
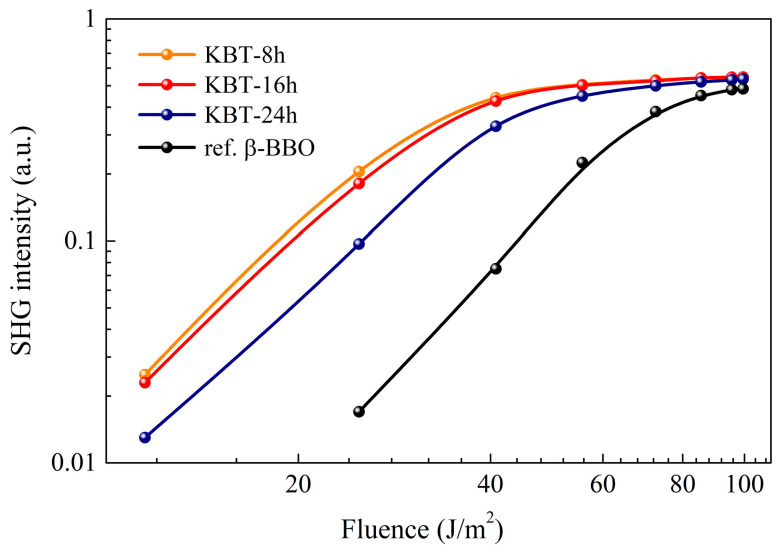
SHG intensity versus laser energy density.

The SHG signal in the function of laser energy density for KBT ceramics and β-BBO reference is depicted in Figure 15. Firstly, the intensity of the detected SHG light of all KBT specimens was nearly identical for higher laser energy densities. Furthermore, a similar SHG characteristic was observed for the non-linear optical β-BBO crystal, indicating the universality of materials irradiated with an intense laser beam.

Secondly, some discrepancies appeared at lower laser energy densities. The most intense SHG signals were generated for the KBT materials with rapid and moderate milling (8 h and 16 h). For the KBT-24h specimen, an SHG signal deficiency indicates that milling time is crucial for KBT ceramics, and excessively minute crystallite sizes deteriorate its phase-matching properties.

**Figure 16:**
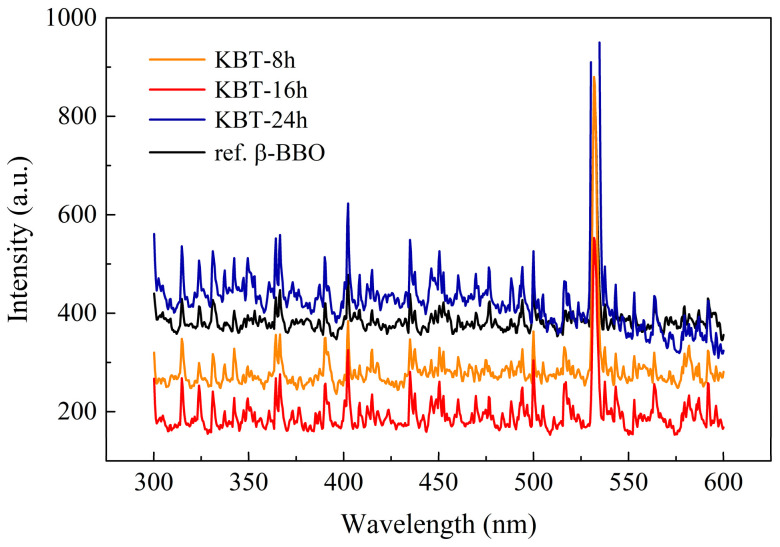
Second-harmonic generation (λ_SHG_ = 532 nm) from the KBT ceramics.

As was mentioned above, the meticulous UV-VIS spectra analysis revealed the nature of the SHG signal (Figure 16). All KBT specimens provided green light (λ_SHG_ = 532 nm) as excited by the fundamental near-infrared radiation (λ_N-IR_ = 1064 nm), thus confirming the spectroscopic relationship ω_N-IR_ = 2·ω_SHG_.

Therefore, it can be concluded that KBT ceramics generate a fairly intense SHG light corresponding to well-known NLO materials, e.g., single-crystal β-BBO. However, the KBT crystallite sizes resulting from the milling time significantly influence the SHG signal intensity.

The above effect was reported, among others, by Ebothe et al. [89], where the critical grain size was examined, for which the SHG signal substantially decreased or completely disappeared.

The last point considers phase matching, which is extremely important for the NLO materials in crystal form. Generally, in monocrystals, the spatial orientation of crystallographic planes is able to optimize the SHG signal. For polycrystalline materials, such as KBT ceramics, the SHG signal detection is generally possible due to the stochastic arrangement of crystallites in the sample volume. Therefore, KBT exhibits a bright glow with a 532 nm wavelength, regardless of spatial orientation.

## 4. Conclusions

The dielectric and non-linear optical properties of bismuth titanate potassium ceramics (KBT) were discussed. For this purpose, a series of complementary investigations were carried out using structural methods (SEM/EDXS, XRD), calorimetry (DSC), and numerous spectroscopies (FT-MIR, FT-FIR, Raman, and EIS). The spectroscopic studies were also compared and explained as part of the DFT calculations. Moreover, the SHG technique showed luminescence centers in the KBT ceramics excited by electromagnetic radiation.

The electronic band structures and density of states (DOSs) of KBT were evaluated using the CASTEP code. The energy gap of 2.46 eV and the direct band-gap transition (*Γ* → *Γ*) satisfactorily correspond to the experimental results.

There is no KBT structural disintegration up to 600 °C, as proved in DSC studies. In contrast, the FT-MIR and FT-FIR results confirmed the thermal and structural stability of KBT ceramics at low temperatures (15–298 K), implying their versatile applications.

The KBT material exhibits peculiar dielectric properties. An EIS study showed a characteristic maximum at *T*_m_ shifting towards higher temperatures with milling time. Indeed, the estimated *T*_m_ temperatures are 343, 352, and 374 °C for KBT-8h, KBT-16h, and KBT-24h, respectively.

Moreover, a significant *ε* enhancement was found for KBT-24h in the whole frequency range. For example, *ε*_R_ (*f* = 1 kHz) at room temperature and *T*_m_ were 24% and 86% higher than for the KBT-16h specimen, indicating that the dielectric parameters increase firmly above a specific milling threshold.

KBT ceramics also reveal non-linear optical properties, being sensitive to excitation by electromagnetic waves. Furthermore, the SHG signal intensity depends on the KBT crystallite sizes and is characterized by a shallow fundamental beam activation threshold compared to β-BBO (see Figure 15). The above benefit designates the KBT material as a near-infrared radiation sensor in the 800–1500 nm range due to a direct IR conversion into visible light (see Figure 14).

In summary, the KBT dielectric and optical properties can be readily adjusted by appropriate selection of crystallite size. Notably, one should emphasize that the lead-free KBT ceramics improved by us have shown a meaningful increase in the *ε*_R_ parameter and interesting non-linear optical properties and can be substitute commercial analogs for PbTiO_3_ and Na_0.5_Bi_0.5_TiO_3_ that are safe for environment.

## Figures and Tables

**Table 1:** The EDXS composition (relative molar fractions) of KBT ceramics.

Specimen	Ti/K	Ti/Bi	K/Bi
KBT-8h	2.05(4)	1.69(3)	0.82(4)
KBT-16h	2.01(3)	1.68(7)	0.83(8)
KBT-24h	2.04(1)	1.73(2)	0.84(8)

**Table 2:** Refined cell parameters and average crystallite sizes (*D*) of the K_0.5_Bi_0.5_TiO_3_ phase in lead-free ceramics at room temperature.

Sample	*a* (Å)	*c* (Å)	*c*/*a*	*V* (Å^3^)	*D* (Å)
KBT-8h	3.9233(3)	3.9849(7)	1.015	61.34	397
KBT-16h	3.9290(1)	3.9783(2)	1.012	61.41	380
KBT-24h	3.9220(3)	3.9966(7)	1.019	61.47	304

**Table 3:** KBT crystallographic data for a 2 × 2 × 2 supercell after DFT optimization.

Atom	Wyckoff Position	*x*	*y*	*z*
K(1)	2*a*	0	0	0
K(2)	2*c*	0	1/2	0
Bi(3)	2*b*	1/2	1/2	0
Bi(4)	2*c*	0	1/2	1/2
O(5)	4*d*	1/4	0	0.25525
O(6)	4*d*	0	1/4	0.25525
O(7)	4*e*	1/4	1/2	0.25525
O(8)	4*e*	1/4	1/2	0.75525
O(9)	8*f*	1/4	1/4	0.01220
Ti(10)	8*f*	1/4	1/4	0.23880

**Table 4:** The selected bond lengths and bond angles of KBT in 2 × 2 × 2 supercell for initial and equilibrium surroundings.

Bonds (Å)	Initial State	Equilibrium	Angles (deg)	Initial State	Equilibrium
K1–K4	5.597	5.600	* Ti36–O24–Bi5	92.72	99.01
K4–O16	2.829	2.876	* K4–Bi8–K7	90.00	95.67
K4–O14	2.769	2.742	* K6–O24–Bi5	91.22	88.21
* K4–Bi3	4.000	3.608	Ti34–O20–Ti38	172.31	161.12
* Ti36–O24	1.962	1.886	O24–Ti36–O16	172.31	172.22
K7–Ti36	3.468	3.422	K1–K4–K1	91.23	90.00
Bi8–K4	3.915	3.930	* Ti36–Bi8–O28	32.59	41.57
* Bi3–O10	2.769	2.338	* O10–Bi3–O14	87.58	110.91
* Ti36–O11	1.962	1.797	O14–K4–Bi2	134.99	134.27
* Bi3–O13	2.839	3.329	Ti36–O15–K7	92.72	88.92

* denotes the most significant deviations.

**Table 5:** Relative permittivity at room temperature (*ε*_RT_) and maximum relative permittivity (*ε*_m_) at *T*_m_ for selected frequencies of K_0.5_Bi_0.5_TiO_3_ (KBT), Na_0.5_Bi_0.5_TiO_3_ (NBT), and PbTiO_3_ (PTO).

Ceramics	1 kHz		100 kHz		1 MHz	
	*ε*_RT_	*ε*_m_	*ε*_RT_	*ε*_m_	*ε*_RT_	*ε*_m_
KBT-8h	844 ± 2	8854 ± 2	657 ± 2	6368 ± 2	594 ± 2	5825 ± 2
KBT-16h	867 ± 2	8346 ± 2	636 ± 2	5273 ± 2	570 ± 2	4708 ± 2
KBT-24h	1074 ± 2	15,559 ± 2	805 ± 2	7453 ± 2	712 ± 2	6502 ± 2
KBT [45]	773	6118	571	3026	532	2708
KBT [49]	1112	5116	881	4537	779	4335
NBT [87]	532	7481	480	4101	461	3869
PTO [54]	1100	4200	700	2300	300	1170

## Data Availability

Data are contained within the article and Supplementary Materials.

## Supplementary Materials

The following supporting information can be downloaded at https://www.mdpi.com/article/10.3390/ma17092089/s1. References [90,91] are cited in the Supplementary Materials.
