# Supplementary material for: Thermal Stability and Non-Linear Optical and Dielectric Properties of Lead-Free K0.5Bi0.5TiO3 Ceramics
Source: Materials (Basel). 2024 Apr 29;17(9):2089. doi: 10.3390/ma17092089 (PMC11084477; doi:10.3390/ma17092089)
Supplement: Supplementary file 1 [file materials-17-02089-s001.zip › materials-2943539-supplementary.pdf]

**Supplementary Materials**

# Thermal Stability and Non-Linear Optical and Dielectric Properties of Lead-Free $K_{0.5}Bi_{0.5}TiO_3$ Ceramics

**Piotr Czaja [1,*], Elżbieta Szostak [2,*], Joanna Hetmańczyk [2], Piotr Zachariasz [3], Dorota Majda [2], Jan Suchanicz [4], Małgorzata Karolus [5], Dariusz Bochenek [5], Katarzyna Osińska [5], Jarosław Jędryka [6], Andriy Kityk [6] and Michał Piasecki [7]**

1 Institute of Technology, University of the National Education Commission, Podchorążych 2, 30-084 Krakow, Poland
2 Faculty of Chemistry, Jagiellonian University, Gronostajowa 2, 30-387 Krakow, Poland; joanna.hetmanczyk@uj.edu.pl (J.H.); majda@chemia.uj.edu.pl (D.M.)
3 Center for Hybrid Microelectronics and LTCC, Łukasiewicz Research Network—Institute of Microelectronics and Photonics, Zabłocie 39, 30-701 Krakow, Poland; piotr.zachariasz@imif.lukasiewicz.gov.pl
4 Department of Mechanical Engineering and Agrophysics, University of Agriculture in Krakow, Balicka 120, 31-120 Krakow, Poland; jan.suchanicz@urk.edu.pl
5 Faculty of Science and Technology, Institute of Materials Engineering, University of Silesia in Katowice, 75 Pułku Piechoty 1a, 41-500 Chorzow, Poland; malgorzata.karolus@us.edu.pl (M.K.); dariusz.bochenek@us.edu.pl (D.B.); katarzyna.osinska@us.edu.pl (K.O.)
6 Faculty of Electrical Engineering, Czestochowa University of Technology, Armii Krajowej 17, 42-201 Czestochowa, Poland; jaroslaw.jedryka@pcz.pl (J.J.); andriy.kityk@univie.ac.at (A.K.)
7 Institute of Physics, Jan Dlugosz University, Armii Krajowej 13/15, 42-200 Czestochowa, Poland; m.piasecki@ujd.edu.pl
* Correspondence: piotr.czaja@up.krakow.pl (P.C.); szostak@chemia.uj.edu.pl (E.S.)

## A. Thermal stability

The chemical stability of the $K_2CO_3$, $TiO_2$, and $Bi_2O_3$ precursors was examined before synthesizing $K_{0.5}Bi_{0.5}TiO_3$ ceramics. Thermogravimetric analysis showed that titanium (IV) and bismuth (III) oxides are stable in the temperature range of 25 ÷ 1000 °C, while potassium carbonate starts to release the absorbed water molecules at 100 °C (Figure S1). Heating anhydrous $K_2CO_3$ above 800 °C enforces its decomposition, which is still incomplete at 1000 °C. The $K_2CO_3$ findings are consistent with the literature reports [S1, S2], indicating the potassium carbonate decomposition temperature under air is about 891 ÷ 896 °C and strongly depends on the heating rate. Thus, the TG analysis was considered when fixing the synthesis conditions of KBT ceramics.

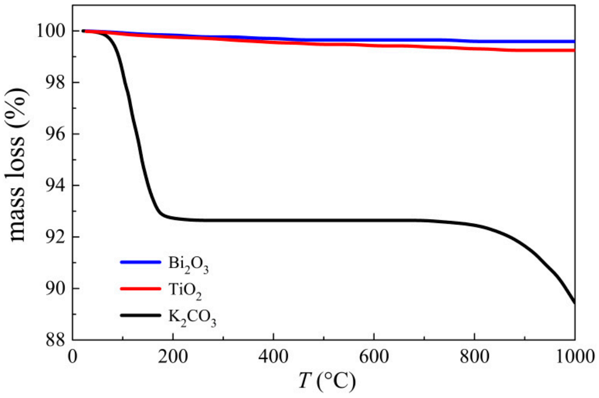


**Figure S1**. Thermogravimetric analysis of $K_2CO_3$, $TiO_2$, and $Bi_2O_3$ at ambient conditions.

**B. Structural characterization**

Figure S2 shows the X-ray diffraction patterns for precursors in the 2θ range of 20º ÷ 60º. XRD data matches the following crystallographic cards: ICDD PDF4+; 00-004-0477 for $TiO_2$, ICDD PDF4+; 01-072-0398 for $Bi_2O_3$, and ICDD PDF4+; 04-014-3875 for $K_2CO_3$, respectively.

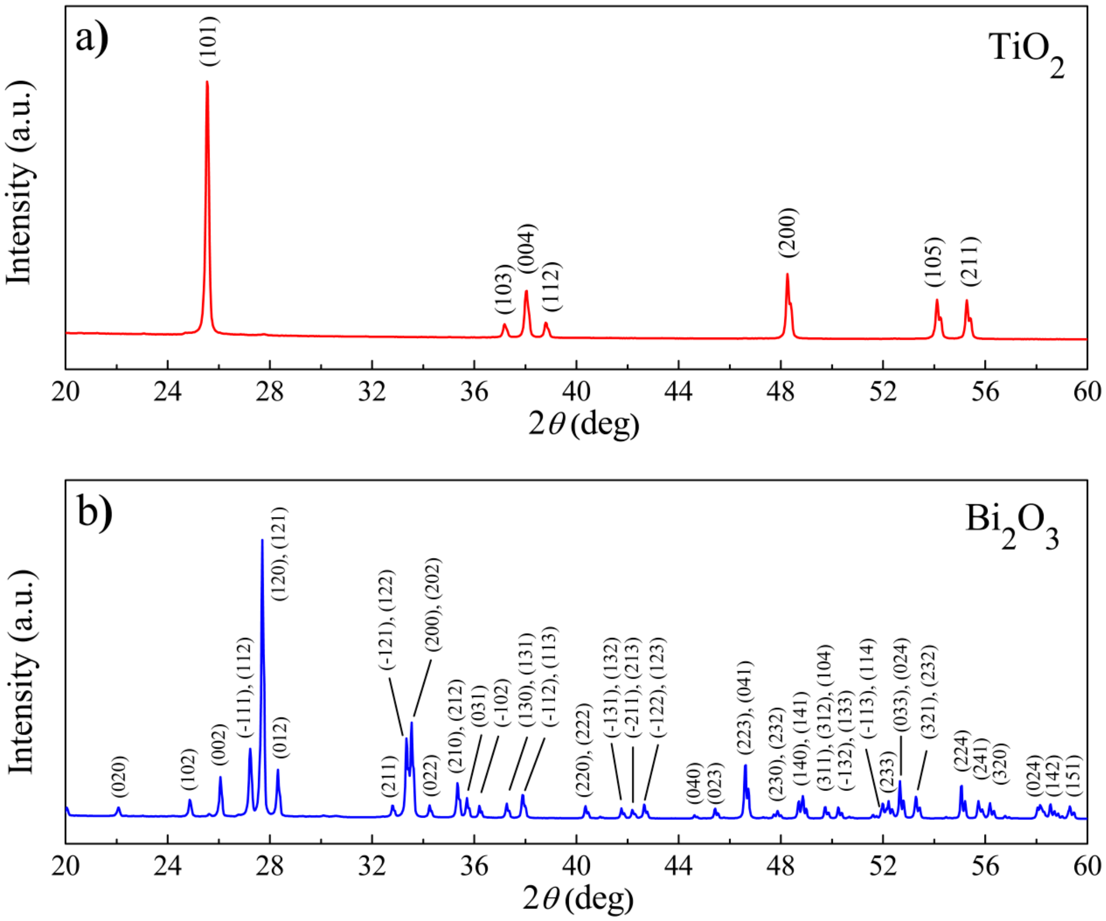

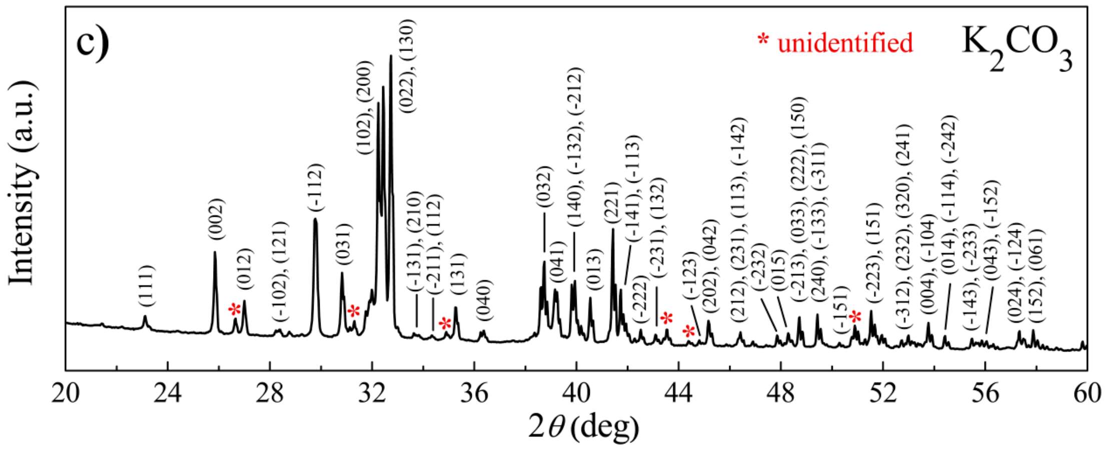


**Figure S2**. X-ray diffraction patterns of (**a**) tetragonal $TiO_2$ and monoclinic (**b**) $Bi_2O_3$, (**c**) $K_2CO_3$ precursors.

X-ray diffraction patterns collected for $TiO_2$ (Fig. S2a) and $Bi_2O_3$ (Fig. S2b) precursors show pure phases; nevertheless, it is possible to recognize some unknown lines for $K_2CO_3$ (Fig. S2c).These results should be considered when fabricating KBT since a foreign admixture can modify calcined or sintering temperature.

## C. Optical vibrational spectroscopy

**Table S1**. Experimental and theoretical vibrational bands for 2x2x2 supercell of KBT.

| **Experiment** | | **DFT calculations** | | | |
|---|---|---|---|---|---|
| **$\nu_{IR}$ [cm$^{-1}$]** | **$\nu_{RS}$ [cm$^{-1}$]** | **ν [cm$^{-1}$]** | **$\nu_{IR}$ [cm$^{-1}$]** | **$\nu_{RS}$ [cm$^{-1}$]** | **Remarks** |
| 819 | 819 | 816 812 802 802 748 | | 803 | $\nu_s$(Ti–O) stretching |
| 656 | 638 | 698 698 618 609 | | 699<br>618 | $\omega$(O–Bi–O) wagging<br>$\nu_s$(Ti–O–Ti) stretching |
| 564 | | 595 588 588 579 579 | 578<br>507 | | $\nu_{as}$(Ti–O–Ti)<br>$\nu_s$stretching |
| 540 | 528 | 540 533 508 508 503 496<br>491 484 481 476 476 467<br>464 462 447 447 444 438<br>438 435 429 | | 537<br>493<br>435 | $\delta$(O–Ti–O) bending<br>deformation modes<br>$\nu_{as}$(Ti–O–Ti)<br>$\nu_s$stretching |
| 410 | | 404 401 401 400 384 383<br>383 372 | 383 | | skeletal bending<br>deformations |
| 389 | | 367 367 360 | 358 | | |
| | 343 | 348 348 344 344 343 342<br>333 333 332 322.7 327.1<br>327 327 | | 360<br>301 | |
| 324 | | 308 304 300 300 299.7<br>295 295 295 282 275 271<br>266 261 256 256 255 | 303 | | skeletal bending<br>deformations |
| 275 | 273 | 240 231 230 230 | 230 | 230 | skeletal bending<br>deformations |
| 228 | | 183 183 180 174 174 170<br>169 168.6 168 168 166<br>163 162 162 159 | 184 | | torsion modes |
| | 206 | 207 204 201 201 197 | | 197 | torsion modes |
| 195 | | | | | |
| | 142 | 149 149 148 147 143 143<br>116 | | 114 | |

| | | | |
|---|---|---|---|
| | 108 108 103 91 86 84 84 | 107 | lattice modes ($\nu_L$) |
| 66 | 57 57 47 47 | | |
| | 0 0 0 | | acoustic modes |

## References


[S1] Zhang, J.; Qi, M.; Zhang, G.; Hu, H.; Xie, L.; Ma, X. Co - production of hydrogen and fibrous carbons by methane decomposition using $K_2CO_3$/carbon hybrid as the catalyst, *International Journal of Hydrogen Energy* **2017**, 42, 11047 – 11052, https://doi.org/10.1016/j.ijhydene.2017.03.113.

[S2] Lehman, R.L.; Gentry, J.S.; Glumac, N.G. Thermal stability of potassium carbonate near its melting point, *Thermochimica Acta* **1998**, 316(1), 1 – 9, https://doi.org/10.1016/S0040-6031(98)00289-5.
